# Supplementary material for: Resolving the full spectrum of human genome variation using Linked-Reads
Source: Genome Res. 2019 Apr;29(4):635–45. doi: 10.1101/gr.234443.118 (PMC6442396; doi:10.1101/gr.234443.118)
Supplement: Supplemental Material [file supp_gr.234443.118_Supplemental_longranger-2.2.zip › longranger-2.2.tar.bz2!/lr2.2-public/lib/stan/cmdstan/stan/doc/api/html/agrad_2rev_2functions_2modified__bessel__first__kind_8hpp_source.html]

Stan: src/stan/agrad/rev/functions/modified\_bessel\_first\_kind.hpp Source File


|  |  |
| --- | --- |
|  | Stan  2.5.0  probability, sampling & optimization |


- Main Page
- Related Pages
- Namespaces
- Classes
- Files

- File List
- File Members

All Classes Namespaces Files Functions Variables Typedefs Enumerations Enumerator Friends Macros Pages

- src
- stan
- agrad
- rev
- functions

modified\_bessel\_first\_kind.hpp

Go to the documentation of this file.

1 #ifndef STAN\_\_AGRAD\_\_REV\_\_FUNCTIONS\_\_MODIFIED\_BESSEL\_FIRST\_KIND\_HPP

2 #define STAN\_\_AGRAD\_\_REV\_\_FUNCTIONS\_\_MODIFIED\_BESSEL\_FIRST\_KIND\_HPP

3

4 #include <stan/agrad/rev/var.hpp>

5 #include <stan/agrad/rev/internal/dv\_vari.hpp>

6 #include <stan/math/functions/modified\_bessel\_first\_kind.hpp>

7

8 namespace stan {

9  namespace agrad {

10

11  namespace {

12

13  class modified\_bessel\_first\_kind\_dv\_vari : public op\_dv\_vari {

14  public:

15  modified\_bessel\_first\_kind\_dv\_vari(int a, vari\* bvi) :

16  op\_dv\_vari(stan::math::modified\_bessel\_first\_kind(a, bvi->val\_), a, bvi) {

17  }

18  void chain() {

19  bvi\_->adj\_ += adj\_ \* (-ad\_ \* stan::math::modified\_bessel\_first\_kind(ad\_, bvi\_->val\_) / bvi\_->val\_ + stan::math::modified\_bessel\_first\_kind(ad\_ - 1, bvi\_->val\_));

20  }

21  };

22  }

23

24  inline var modified\_bessel\_first\_kind(const int& v,

25  const var& a) {

26  return var(new modified\_bessel\_first\_kind\_dv\_vari(v, a.vi\_));

27  }

28

29  }

30 }

31 #endif

dv\_vari.hpp

modified\_bessel\_first\_kind.hpp

stan::agrad::modified\_bessel\_first\_kind

fvar< T > modified\_bessel\_first\_kind(int v, const fvar< T > &z)

**Definition:** modified\_bessel\_first\_kind.hpp:15

stan::agrad::var::vi\_

vari \* vi\_

Pointer to the implementation of this variable.

**Definition:** var.hpp:40

var.hpp

stan::agrad::var

Independent (input) and dependent (output) variables for gradients.

**Definition:** var.hpp:27

stan::math::modified\_bessel\_first\_kind

T2 modified\_bessel\_first\_kind(const int v, const T2 z)

**Definition:** modified\_bessel\_first\_kind.hpp:39


---

[ Stan Home Page ]

*© 2011–2014,
Stan Development Team.*
